# Model-Based Design of Experiments for Temporal Analysis of Products (TAP): A Simulated Case Study in Oxidative Propane Dehydrogenation

**DOI:** 10.1021/acs.iecr.3c03418

**Published:** 2024-03-11

**Authors:** Adam Yonge, Gabriel S. Gusmão, Rebecca Fushimi, Andrew J. Medford

**Affiliations:** † School of Chemical & Biomolecular Engineering, Georgia Institute of Technology, Atlanta, Georgia 30332, United States; ‡ Catalysis and Transient Kinetics Group, Idaho National Laboratory, Idaho Falls, Idaho 83415, United States

## Abstract

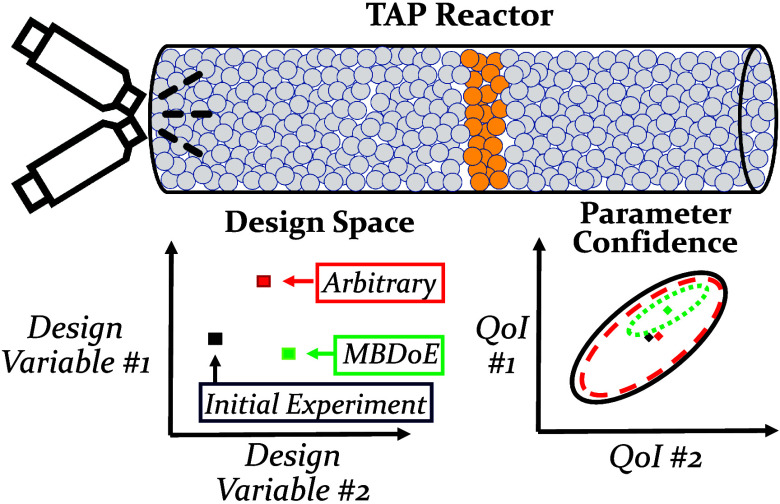

Temporal analysis of products (TAP) reactors enable experiments that probe numerous kinetic processes within a single set of experimental data through variations in pulse intensity, delay, or temperature. Selecting additional TAP experiments often involves an arbitrary selection of reaction conditions or the use of chemical intuition. To make experiment selection in TAP more robust, we explore the efficacy of model-based design of experiments (MBDoE) for precision in TAP reactor kinetic modeling. We successfully applied this approach to a case study of synthetic oxidative propane dehydrogenation (OPDH) that involves pulses of propane and oxygen. We found that experiments identified as optimal through the MBDoE for precision generally reduce parameter uncertainties to a higher degree than alternative experiments. The performance of MBDoE for model divergence was also explored for OPDH, with the relevant active sites (catalyst structure) being unknown. An experiment that maximized the divergence between the three proposed mechanisms was identified and provided evidence that improved the mechanism discrimination. However, reoptimization of kinetic parameters eliminated the ability to discriminate between models. The findings yield insight into the prospects and limitations of MBDoE for TAP and transient kinetic experiments.

## Introduction

Although heterogeneous catalysts have been studied for well over a century, there are many remaining challenges to systematically understand the reaction mechanisms and active sites that govern their behavior in chemical reactors.^1^ These challenges are a result of many factors, including the complexity of materials, various length scales involved in catalytic processes, and time scales over which catalytic events take place.^2,3^ By quantitatively understanding materials and their associated kinetics, we can rationally identify and optimize promising catalysts. A wide range of computational and experimental methods have been developed to explore the kinetics of heterogeneous catalysts. At the atomic scale, the electronic structure of individual atoms and molecules can be investigated with computational simulations, surface science experiments are commonly used to study well-defined surfaces, and catalyst particles or packed-bed reactors (PBRs) can be utilized to provide insight into the reactor-scale behavior of catalysts. However, the “pressure gap” and “materials gap” create challenges in connecting behavior between these scales.^4−10^

Even with the availability of these tools, it can be challenging to derive strong mechanistic insights into catalytic materials due to the presence of experimental, parametric, and structural uncertainties.^11^ These uncertainties can easily lead to confidence intervals of quantities of interest (QoI) that span several orders of magnitude.^12^ For this reason, much effort has been made over the past decade to identify and reduce these sources of uncertainty. For example, Heyden and co-workers have propagated density functional theory (DFT) free-energy uncertainties, which are known to be sensitive to the choice of the exchange-correlation functional, to the QoIs in microkinetic models (e.g., turnover frequencies, apparent activation energy) to help discriminate between competing proposed reaction pathways.^12,13^ Nørskov and co-workers have also investigated the role of exchange-correlation uncertainty in catalyst screening by propagating the model uncertainties from the BEEF–vdW functional to microkinetic models for screening catalysts for the ammonia synthesis and syngas conversion reactions.^14,15^ Vlachos and co-workers have similarly progressed the field through their study of scaling relationship errors on selectivity predictions, as well as uncertainty-based analysis of experimental data and coverage effects.^16−18^ From these studies and others, various formalisms and software packages have also been developed to quantify and evaluate the impact of uncertainty within the field of catalysis and reaction modeling.^19−22^

**Figure 1:**
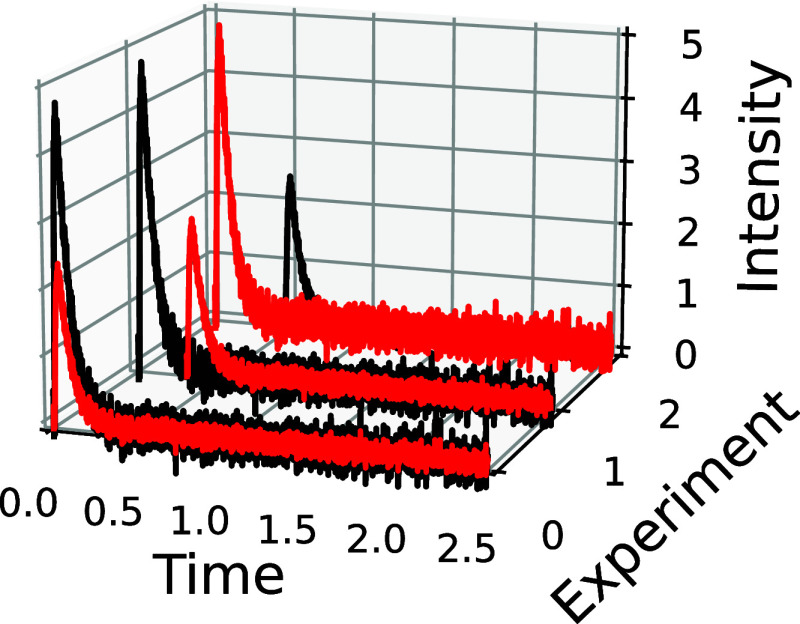
Illustration of pump–probe TAP experiments, where pulses of two reactants (red and black) are pulsed with varying intensities and delays between different experiments.

One challenge in catalysis is that it is often difficult or impossible to directly extract intrinsic kinetic parameters from experimental data sets. Steady-state kinetic measurements are typically sensitive to only a few parameters and are therefore known to be prone to overfitting when complex kinetic models are used.^23,24^ Surface science techniques allow the measurement of specific adsorption energies and reaction barriers, but they typically require well-defined surfaces that may differ from complex nanoparticles used in real applications, and the adsorption and reaction barriers that are extracted are also prone to uncertainty comparable to calculated quantities.^25−27^ Transient kinetic experiments can overcome some of these challenges because they are sensitive to a larger number of elementary processes compared to steady-state experimental approaches. In recent years, there has been renewed interest in the use of transient experiments to explore catalytic materials, including steady-state isotopic transient kinetic analysis (SSITKA) and spectrokinetics.^28−34^ An additional transient kinetic experimental approach is the temporal analysis of products (TAP) reactor. The TAP approach uses a small (approximately 4 cm long) PBR operating under ultrahigh vacuum (UHV) conditions. A TAP experiment consists of a series of rapid nanomolar pulses of reactants, closely related to molecular beam experiments.^35^ A pulse valve introduces these molecular pulses at the entrance of the reactor, and the molecules diffuse to the outlet where a mass spectrometer is located to detect the outlet flux of all gases. The reactor can be used with complex industrial catalysts and exhibits a well-defined transport regime, i.e., Knudsen diffusion, which aids in the deconvolution of transport and kinetics.^36−39^ The TAP approach has also been used for pump–probe experiments, which can provide even more control over which elementary steps are probed by pulsing different reactants with time delays.^36,40−42^ Altering the reactant feed varies the surface coverage, which, in turn, can alter the activity of various elementary processes. An illustration of the experimental conditions used in a pump–probe TAP experiment is shown in Figure 1. While TAP experiments offer information-rich data sets, complex data analysis and model fitting techniques are required to extract kinetic information, and the resulting models and parameters still have a broad range of uncertainties associated with them.^43−45^

Gathering additional experimental data is one route to reduce uncertainty in fitted models, and this is well-suited to TAP experiments, where pulses can be collected on the second time scale (with up to 10 000 pulses at different conditions collected within a single day), and experimental conditions can be relatively easily varied.^46^ For example, Wang et al. used fixed variations in the pulse delay of isotopic oxygen species to better understand the active oxygen species in the oxidative coupling of methane process.^47^ These pump–probe experiments can help target the expression of elementary processes that might be masked under alternative conditions. At present, additional TAP experiments are typically selected on the basis of a user’s chemical intuition, which is a qualitative approach that may not yield optimal results.

The model-based design of experiments (MBDoE) has been studied for the last few decades and offers a quantitative means to guide experimental selection based on improved precision and mechanism discrimination.^46^ MBDoE can also help address issues with respect to structural uncertainty, which arises due to uncertainty in the elementary steps and the number of active sites involved in the reaction mechanism.^48−50^ MBDoE has been applied to some transient kinetic examples, but the complexity of the reaction mechanisms and reactor models studied is lower than that of typical TAP experiments.^51−53^

In this study, we outline the theoretical terms of MBDoE in terms of TAP reactor experiments and apply the methodology to a synthetic oxidative propane dehydrogenation (OPDH) process. We identify potential limitations of this framework and show how it can be modified to target the reduction of parametric uncertainty in kinetic parameters. We also explore the structural uncertainties present in kinetic mechanisms and active site structures through the MBDoE for divergence. Some studies of mechanism discrimination have previously been performed for TAP experiments, but no implementations were introduced to quantitatively design experiments.^54^ Mechanistic uncertainty is also explored through active site configuration variations in the synthetic OPDH process. The challenges of pairing mechanism discrimination with optimization are discussed as well as prospects for making the MBDoE for precision and discrimination more practical.

## Methodology

The equations associated with the modeling of the TAP reactor and MBDoE have been thoroughly outlined in previous publications.^44,46^ For clarity, the necessary equations for this work are introduced in the following subsections. The proposed workflows for precision and discrimination are also introduced as well as the OPDH mechanisms used as a case study. All simulations and optimizations were performed using the open source Python package TAPsolver.^44^

### TAP PDEs and Uncertainty Quantification

The fundamental TAP reactor equation consists of (Knudsen) diffusion and a reaction term (generalizing all reactions involving the gas species). This equation is written as
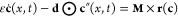
1where the void fraction is defined as ε, the concentrations of species *i* in the system are defined by vector ***c*** = [*c*_*i*_]^*T*^, with ∂_*t*_***c*** = ***ċ*** and ∂_*x*_***c*** = ***c***′ representing the time (*t*) and spatial derivatives (*x*) of the concentrations, respectively, ***d*** = [*D*_*i*_]^*T*^ defines the Knudsen diffusivity for each gas, ***M*** represents the stoichiometry matrix of the reaction system, ***r*** is a vector with rates of individual reactions, and ⊙ represents the element-wise product.

The reaction rate vector is computed from a kinetic model with rate constants that are obtained from reaction free energies (Δ*G*) and activation energies (*G*^‡^), which are the intrinsic catalytic properties that govern the behavior of the system. The reactor is assumed to be isothermal since reactant pulses are typically on the nanomolar scale, ensuring that heat can be dissipated. The gas concentrations for all species along the reactor are initialized to zero, written as
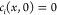
2

The experiment begins by introducing a set of reactant and inert pulses at the entrance of the reactor, mathematically defined as
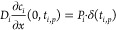
3with *P*_*i*_ and *t*_*i*,*p*_ representing the pulse intensity (or total number of molecules being introduced into the reactor) and pulse delay (or feed time) of gas *i*, respectively. At the exit of the reactor (*L*), a vacuum is applied, and the concentration is zero for the duration of the experiment
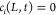
4

Surface species in the catalyst zone of the TAP reactor, represented by *u*_*i*_, follow a similar equation but without diffusive transport. The initial concentration is defined as
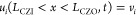
5with *L*_CZI_ and *L*_CZO_ representing the catalyst zone inlet and outlet length, respectively, and *v*_*i*_ representing the explicit initial concentration. Outside of the catalyst zone, the surface concentration is assumed to be zero since an inert material should not interact with the gas species. As noted in the introduction, the outlet flux of each gas is the primary experimental data extracted from the TAP reactor. The outlet flux is defined as
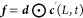
6where ***f*** is the vector of fluxes and *L* is the length of the reactor. When fitting TAP experimental data with PDE solutions, it is also necessary to define the objective function over the time steps (*S*) and multiple experiments (*N*)
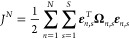
7where **Ω**_*n*,*s*_ is the precision matrix for experiment *n* and time step *s*, i.e., the inverse of the covariance matrix **Σ**_*n*,*s*_, and **ε**_*n*,*s*_ is the model residual
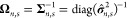
8
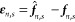
9with **σ̂**_*n*,*s*_ being the standard deviation of the experimental noise at time *s*, ***f̂***_*n*,*s*_ representing the simulated outlet flux, and ***f***_*n*,*s*_ standing for the experimental outlet flux.

The noise present in the observed data comes primarily from the mass spectrometer and translates into uncertainty in the kinetic parameters. Although signal correlation may be present in TAP outlet fluxes, it is not typically quantified, so we performed the analysis with the assumption of no correlation (making eq 7 a weighted least-squares objective function). This standard deviation is defined in eq 8 as σ**^**_*n*,*s*_ and is approximated as Gaussian, although the standard deviation depends on both time and gas species so that, in principle, it is possible to account for heteroscedastic errors that have been reported for data from mass spectrometers,^43^ though we assume homoscedastic errors in this work. Near the local minima of this objective, the shape of the well can be approximated as quadratic. Calculating the Hessian, which is defined as
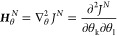
10near this point allows confidence intervals to be extracted following optimization.^55,56^ In eq 10, θ is the kinetic parameter of interest. The confidence intervals can then be extracted from this Hessian through the following equations
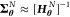
11
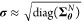
12where **Σ**_θ_^*N*^ is the covariance matrix and **σ** is the vector of standard errors of each fitted parameter due to experimental signal noise. We note that the standard error above is used to represent the confidence intervals discussed later in the text (95% C.I. is equivalent to two times the standard error) and that they assume linearity. Although parameter correlation is not taken into account in **σ**, correlation information can be extracted from the covariance matrix we explicitly evaluate.

### Synthetic Oxidative Propane Dehydrogenation Case Study

We select OPDH as a case study since it is industrially relevant for propylene production, requires understanding of both rate and selectivity, and has mechanistic and active site complexity that provides a rich set of theoretical challenges.^57−59^

The kinetics of the OPDH process have been broadly studied. We base our model parameters and structure on experimental kinetic analyses found in the literature.^60,61^ The Eyring equation is used to define the relationship between the free energy and the rate constant and is written as
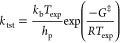
13with *k*_tst_ representing the rate constant, *k*_b_ the Boltzmann constant, *h*_p_ the Planck constant, and *R* the ideal gas constant. The mechanism used is provided as mechanism 1 in Table 1. The mechanism is not composed of elementary steps, which limits the complexity of the analysis and is consistent with the fact that not all elementary processes can be observed from TAP experiments. For example, the reverse reactions in a combustion reaction or some readsorption processes are unlikely to be observed, even with the low pulse intensity and pressure found in TAP. Similar reaction steps to those found in Table 1 have been used in kinetic models for oxidation reactions of other hydrocarbons.^62^

**Table 1:** Gibbs Free Energies of Reaction and Activation for the Synthetic OPDH Model^a^

	step	reaction	(eV)	(eV)
mechanism #1	1		–0.2	0.3
	2		–0.7	1.25
	3		–0.1	0.2
	4		–0.35	1.54
	5		–3.98	1.65
	6		–3.62	1.37
	7		–8	0.1
				
mechanism #2	1	C_3_H_8_ + * ↔ C_3_H_8_*	–0.2	0.3
	2		–0.7	1.25
	3		–0.1	0.2
	4		–0.35	1.54
	5		–3.98	1.65
	6		–3.62	1.37
	7		–8	0.1
				
mechanism #3	1		–0.2	0.3
	2		–0.7	1.25
	3		–0.1	0.2
	4		–0.35	1.54
	5		–3.98	1.65
	6		–3.62	1.37
	7		–8	0.1
	8		0.36	1.45

a In mechanism 1, we include only a single active site for all of the kinetic processes. In mechanism 2, a separate site is included for oxygen (i.e., oxygen and the carbon species do not compete for the same site). In mechanism 3, surface hydrocarbons combust on a separate active site from the remaining kinetic steps.

We also explore the structural uncertainty in this case study, referring to both the uncertainty in the elementary steps involved and the active site(s) on which reactions take place. In the case of OPDH, it is often hypothesized that different reactions occur on different types of active sites.^60,63^ For this reason, we defined two additional multisite variations of the single-site OPDH mechanism (presented in Table 1). These reactions have two separate active sites, where mechanisms 2 and 3 involve the isolation of oxygen adsorption and propane combustion, respectively, as inspired by various hypotheses in the literature.^64^ Notably, this presents a particularly difficult challenge in model discrimination since the elementary steps included and the kinetic parameters are the same between all three models, allowing a specific focus on the question of whether TAP experiments can distinguish between single-site and multisite mechanisms.

### Workflows for MBDoE of TAP Experiments

**Figure 2:**
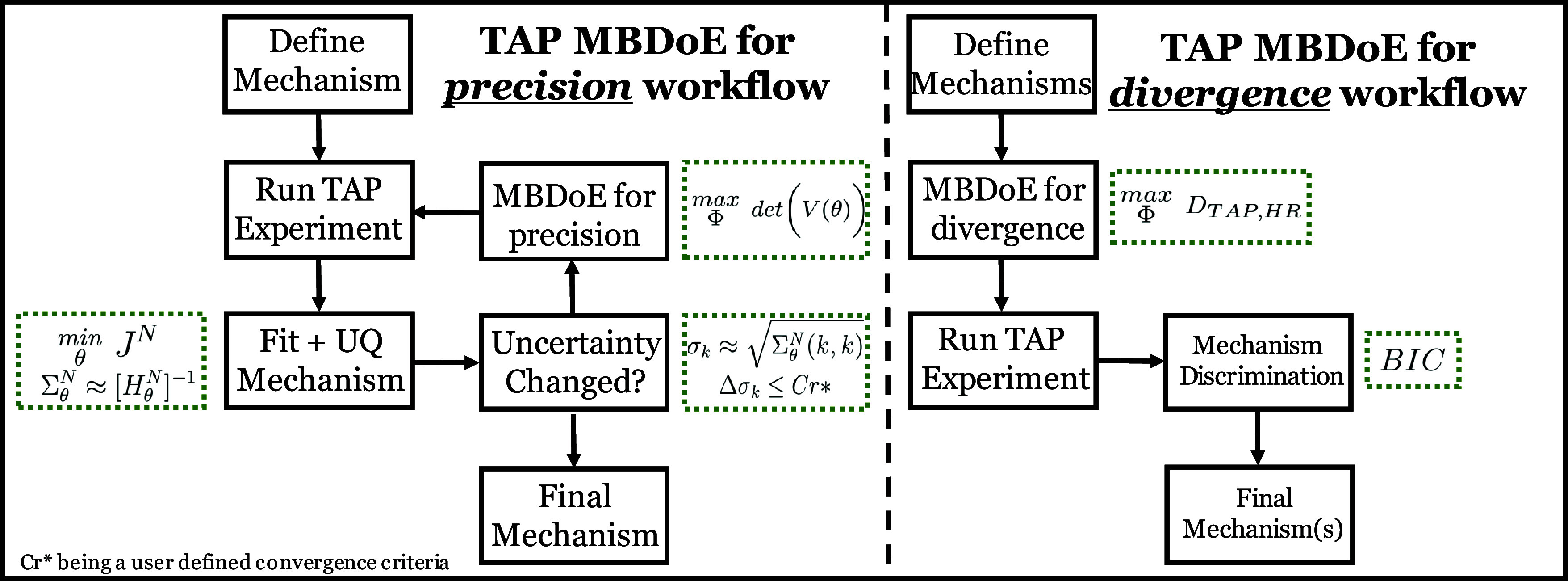
Proposed general workflow for selecting additional TAP experiments through MBDoE for precision as well as the steps for selecting an experiment to maximize divergence.

In this study, we introduce two general workflows for reducing parametric uncertainty (precision) and structural uncertainty (divergence) in kinetic models using TAP experiments. The workflows are outlined in Figure 2. We propose that the user begins with a potential reaction mechanism. In the case of precision refinement, the user first runs an arbitrary TAP experiment. Following the experiment, the initial round of optimization and uncertainty quantification (UQ) should be performed. Next, the optimality criteria should be used to determine the effect the experimental conditions have on parameter identifiability. If high variation is observed, the experiment with the highest value for the criteria should be selected, performed, and reoptimized. This continues until the user has sufficiently reduced the uncertainty, or the predicted optimal criteria begin to experience limited reduction. In the case of divergence, it is assumed that different possible mechanisms and parameters can be defined a priori. From there, the MBDoE for divergence will identify the experiment that maximizes the divergence between the mechanisms, and fitting the different mechanisms to the results of the experiment provides evidence to support mechanism discrimination by comparing an information criterion. Details and variations of these workflows are described in subsequent sections.

We used a grid search to explore the design space, with propane and oxygen intensity, a pulse delay of propane, and reactor temperature as the design parameters. Alternative approaches to optimizing the experiment could be used (i.e., a gradient-based approach), but we chose a grid-based search algorithm due to ease of implementation and parallelization. We chose pulse intensity values of 1, 1.5, and 2 nmol, propane pulse delays of 0, 0.15, 0.3, 0.45, and 0.6 s, and reactor temperatures of 650, 675, and 700 K as the possible values in the grid, leading to 135 potential experiments.

### MBDoE for Precision in TAP

TAP experiments allow flexible specification of initial conditions including pulse intensity, pump/probe delay, surface coverage, and reactor temperature. The feed concentrations (i.e., intensities) can be readily changed in any experiment, providing the experimentalist with strong control over surface coverage variations. The tuning of these parameters allows for the targeted expression of elementary processes in a reactor system, which can be beneficial to fully understand the mechanism and intrinsic kinetic parameters. The understanding of a mechanism is not static and can evolve with the inclusion of new experimental data. Identifying which experiments will yield the most insight into catalytic systems can be complex, and relying on chemical intuition can be inefficient. MBDoE offers a quantitative strategy for the selection of additional experiments that maximize information content.

The first step in the design of experiments is to arrive at an initial guess of the parameters, optimize them, and quantify the associated uncertainties (Figure 2). Using this initial understanding of the model and parametric uncertainty, it is possible to predict the conditions that will reduce the uncertainty the most in future experiments. This is achieved through the use of output sensitivities with respect to parameters of interest over time, or the dynamic sensitivity matrix. The dynamic sensitivity matrix is constructed as
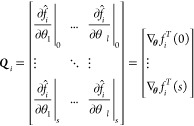
14with *f̂*_*i*_, θ, *l*, and *s* representing the simulated outlet flux, the fitted parameter of interest, the final parameter being considered in the set, and the total number of time steps in the system, respectively. A matrix is constructed for each outlet gas *i* and has a number of columns equal to the number of parameters and a number of rows equal to the number of time steps. The derivatives are evaluated using a finite difference approach with a variation multiplier of 1e–5. These dynamic matrices are then consolidated into Fisher information matrices.^65^ The Fisher information matrix quantifies how informative a new experiment will be, with respect to a proposed kinetic model and the associated parameters. The inverse Fisher information matrix is defined in terms of the dynamic sensitivity matrix as
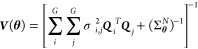
15where Σ_θ_^*N*^ is the covariance matrix of the model (see eqs 7–12), σ_*i*,*j*_ is the outlet flux noise, and *i* and *j* are species in the set of gases *G*. The inverse Fisher information matrix is typically distilled into a single quantitative criterion. Interpreting and comparing scalar values are simpler than comparing matrices. Three criteria for optimality are frequently used in the MBDoE for precision. One is the A optimality, which is a measure of the trace of the information matrix (or total variance)
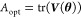
16Another is the *E* optimality, which aims to minimize the largest eigenvalue of the covariance matrix
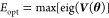
17Finally, *D* optimality is the determinant of the inverse Fisher information matrix (or a product of the standard deviations)
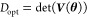
18

When trying to predict an optimal experiment, it is desired to find the experiment that will result in the lowest *A*, *D*, or *E*-value. These minimum values are meant to correlate with the maximum amount of information in the system where each criterion corresponds to a different definition of information.

We first evaluate MBDoE as a strategy to systematically reduce the uncertainty in fitted parameters. We utilize the parameters for mechanism 1, as defined in Table 1 for all data generation in this section. We focus the analysis on a subset of seven parameters (Δ*G*_0_, Δ*G*_1_, Δ*G*_2_, *G*_1_^‡^, *G*_3_^‡^, *G*_4_^‡^, *G*_6_^‡^) selected based on an initial sensitivity analysis that identifies these as the parameters that have the most influence on the model (provided in the Supporting Information). This is consistent with the similar energy scale for these parameters (Δ*G* ∼ (−1, 0) eV, *G*^‡^ ∼ (1, 2) eV), since very low barriers lead to effectively equilibrated reactions and very negative reaction free energies lead to effectively irreversible steps, making it difficult to deduce these parameters from experimental data. For the inverse problems in this work, we fix the excluded parameters to their true value and use initial guesses of −0.3 eV for all adsorption/reaction energies and 1.5 eV for activation energies. In practice, determining these initial guesses would require some prior knowledge, global optimization techniques, and sensitivity analyses. However, here we are primarily concerned with the ability of MBDoE to refine the precision of parameter estimates and restrict our analysis to how additional experimental data improve accuracy and reduce uncertainty on local optimization of kinetic parameters.

We note the challenge of insufficient data for parameter determination. In some instances, parameters will not be sensitive to the experimental data. When parameters are not sensitive to the system but are included in the information matrix, the matrix can become noninvertible and impede experimental design. This is an area of active research that should be explored further.^66^ Here, we avoid the issue by only evaluating parameters that are determined to be sensitive based on the initial optimization and sensitivity analysis.

#### MBDoE for Divergence in TAP

**Figure 3:**
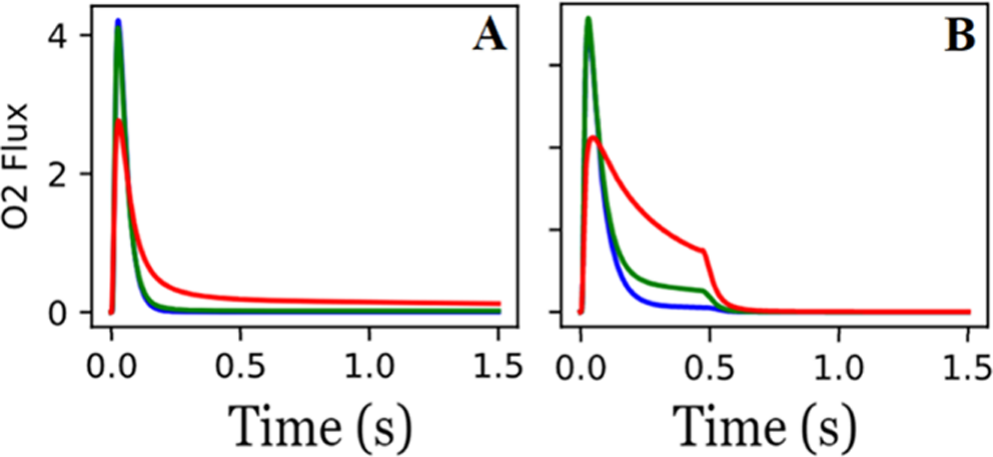
Example of mechanism divergence observed between three potential mechanism (green, blue, and red) simulations at two different TAP experimental conditions (A, B). The two experiments do not equally differentiate the mechanisms, with experiment (B) showing a stronger divergence between the green and blue mechanisms in the tail of the outlet flux.

The goal of MBDoE for model divergence is to identify a crucial experiment that can be used to differentiate between possible models that describe the data, as illustrated in Figure 3. To achieve this, each known model is used to simulate the system over the initial condition grid. At each combination of initial conditions selected, the simulated outlet flux will be available for all gas-phase species for each of the mechanisms being explored. Of the initial conditions explored, the one that maximizes the divergence between the results of the simulations from different models is selected. When there is no consideration of the uncertainty, the divergence criteria can be written in a form derived by Hunter and Reiner and adjusted specifically for TAP as
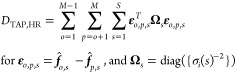
19where *M* is our set of models, *S* is the time step where flux data points were collected, ***f̂*** = {*f̂*_*i*_} is the simulated flux,^67^ and **Ω**_*s*_ is the estimated precision matrix at time *s*.

Discriminating between mechanisms based on qualitative deviations can be challenging and lead to varying results based on the user’s intuition.^68^ For this reason, significant efforts have been made to establish quantitative criteria for selecting between competing mechanisms. We evaluated the quality of a model using the Bayesian information criteria (BIC), which penalizes models with more parameters.^69^ BIC is a commonly used method for selecting between competing mechanisms and is defined as
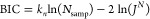
20with *N*_samp_ representing the sample size (number of data points) of the system and *k*_*n*_ representing the number of parameters in the model. BIC parameter penalization increases with the size of the data set, and the values are evaluated with kinetic parameters in the following section. We note that the BIC can be calculated with any set of kinetic parameters, and in the subsequent section and discussion, the BIC with nonoptimized and optimized kinetic parameters are compared.

## Results

### MBDoE for Precision

**Figure 4:**
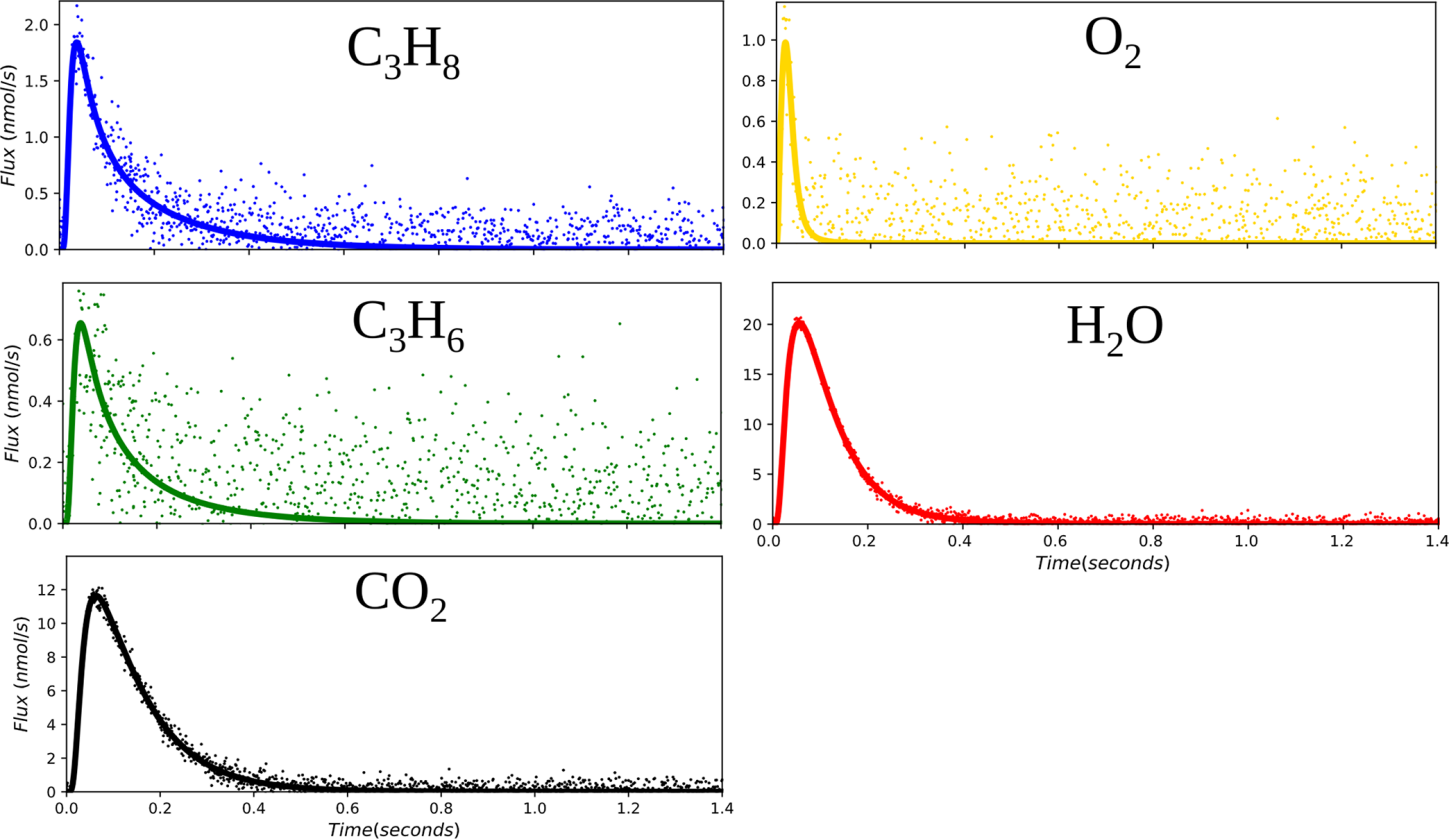
Initial OPDH mechanism fit (model results are represented by each line, while data is represented by the points) to the synthetic experimental data using a reactor temperature of 700 K, a 1 nmol pulse of C_3_H_8_ and O_2_, and no delay between either gas pulse.

To initiate MBDoE, we first need an experiment to establish parameter estimates. A simple choice for an initial experiment is equimolar pulses of propane and oxygen. Copulsing these species simultaneously is selected since it is a natural boundary in the delay space. A reactor temperature of 700 K was selected as a typical temperature for an OPDH reaction. The experiment was simulated at these conditions, and the kinetic parameters of the model were optimized to the data set using standard TAPsolver settings and initial guesses as described in the Methodology section. The fitted model is presented in Figure 4 and the optimized parameters and their 95% confidence intervals are shown in Table 2. The results reveal a strong agreement between most of the fitted parameters and the ground truth values. Most of the fitted parameters are within 0.02 eV of the ground truth, with the exception of the free energy of oxygen adsorption (Δ*G*_1_), which has a more significant error of 0.17 eV. The uncertainty in Δ*G*_1_ is likely the result of a low sensitivity of the parameter to the experimental conditions.

**Table 2:** Actual Values, Initial Guesses, and Values Following Optimization with Simulated Experimental Data Sets 1, 2, and 3 (Defined in Table S2)^a^

	actual	initial		exp. 1		exp. 2		exp. 3		exp. alt.	
	value	value	C.I.	value	C.I.	value	C.I.	value	C.I.	value	C.I.
	–0.20	–0.30	**–**	–0.20	2.73e-3	–0.20	8.17e-4	–0.20	5.77e-4	–0.20	6.14e-4
	–0.70	–0.30	**–**	–0.53	9.51e-2	–0.70	2.25e-2	–0.71	2.19e-2	–0.70	2.67e-3
G_1_^‡^	1.25	1	**–**	1.24	2.57e-2	1.24	5.98e-4	1.25	4.26e-4	1.25	5.04e-4
Δ*G*_2_	–0.10	–0.30	**–**	–0.10	4.40e-2	–0.12	9.27e-3	–0.10	1.03e-2	–0.12	6.73e-3
*G*_3_^‡^	1.54	1.5	**–**	1.54	2.41e-3	1.54	8.49e-4	1.54	6.09e-4	1.54	6.67e-4
*G*_4_^‡^	1.64	1.5	**–**	1.63	3.88e-2	1.63	5.94e-3	1.65	6.55e-3	1.63	5.25e-3
*G*_5_^‡^	1.37	1.5	**–**	1.37	4.51e-2	1.39	9.47e-3	1.37	1.05e-2	1.39	6.79e-3

a The confidence intervals (95%, labeled as the C.I. equal to twice the standard error) of the parameters following optimization are also provided. The values of the alternative approach (labeled exp. alt.) are also presented in the right most column.

The confidence intervals of many parameters are on the order of 0.01–0.1 eV. Given that rates depend exponentially on these free-energy values, it is of interest to systematically reduce the confidence intervals to yield more precise estimates.

The first round of MBDoE can be performed once the initial parameter estimates and uncertainties are established. We perform a grid search over the initial conditions considered and calculate the inverse Fisher information matrices (eq 15). Next, an optimal criterion must be selected. Based on prior literature, we select *D* optimality as the criterion to use, so the optimal experiment is the one with the smallest determinant of the inverse Fisher information matrix.^51,53^ We also explored other criteria and confirmed that *D* optimality is the most effective in this case (see the Supporting Information). Using the *D* optimality criterion, we find that an experiment with 2 nmol propane and oxygen pulses, a propane delay of 0.6 s, and a temperature of 650 K was best in the grid search (a table of the designed experiments is shown in the Supporting Information). We refit the parameters with the data from this new experiment and the data from the first experiment included in the loss function. The new values of the parameters and their confidence are listed in Table 2. The estimates of most parameters remain approximately constant, with the exception of Δ*G*_1_, which exhibits strong agreement with the ground truth and significantly lower error bars after the addition of the new data. The confidence intervals of other parameters also decrease, indicating that the additional experiment indeed improves the accuracy and precision of the estimated parameters.

Following the MBDoE for the precision workflow outlined in Figure 2, we continue with another iteration of the MBDoE to see if the uncertainty can be further reduced. We follow the same grid search approach previously used and find that a similar experiment is identified. However, the delay is selected to be 0.15 s instead of 0.6 s. We add the data from this additional experiment to the loss function and provide the parameter values and confidence intervals after optimization in Table 2. Both the parameter estimates and confidence intervals are relatively similar after the inclusion of this additional data set. Some confidence intervals are slightly reduced, while others see a slight increase, which could be a result of variations in the confidence intervals of other parameters. The approximately static parameter estimates and confidence intervals suggest that the MBDoE workflow will see limited additional improvement and that further experiments are unlikely to have as dramatic an impact on the uncertainty.

### MBDoE for Model Discrimination

The use of MBDoE for model discrimination follows a different structure and set of assumptions from MBDoE to improve parameter precision. We use the mechanisms and associated parameters found in Table 1, but in this case, the goal is to investigate whether TAP experiments can be used to distinguish these subtly different mechanisms. To create a more realistic scenario, we add a small amount of Gaussian noise (σ = 0.05 eV) to the parameters used to generate the entire experimental data set (i.e., identical parameter values are used to generate the synthetic experimental data at all reactor conditions). These minor deviations are meant to create a more realistic scenario since they create a slight discrepancy between the “true” parameters used to create the synthetic data and the parameters used in simulations for model discrimination. In other words, we assume that the models used to discriminate between different kinetic behaviors do not have the exact parameters that govern the experimental data. In this scenario, we assume that three possible mechanisms and associated rate constants have been identified from another experimental approach, calculations, or the literature, and the goal is to determine the conditions of the TAP experiment that lead to the greatest divergence between the results of experiments for different mechanisms. In principle, this leads to three different “ground truths”, corresponding to scenarios where each of the possible mechanisms is the true one. Here, we focus on the case where mechanism 2 is the “ground truth” since it yielded results where model discrimination was again nontrivial (see the Supporting Information).

**Figure 5:**
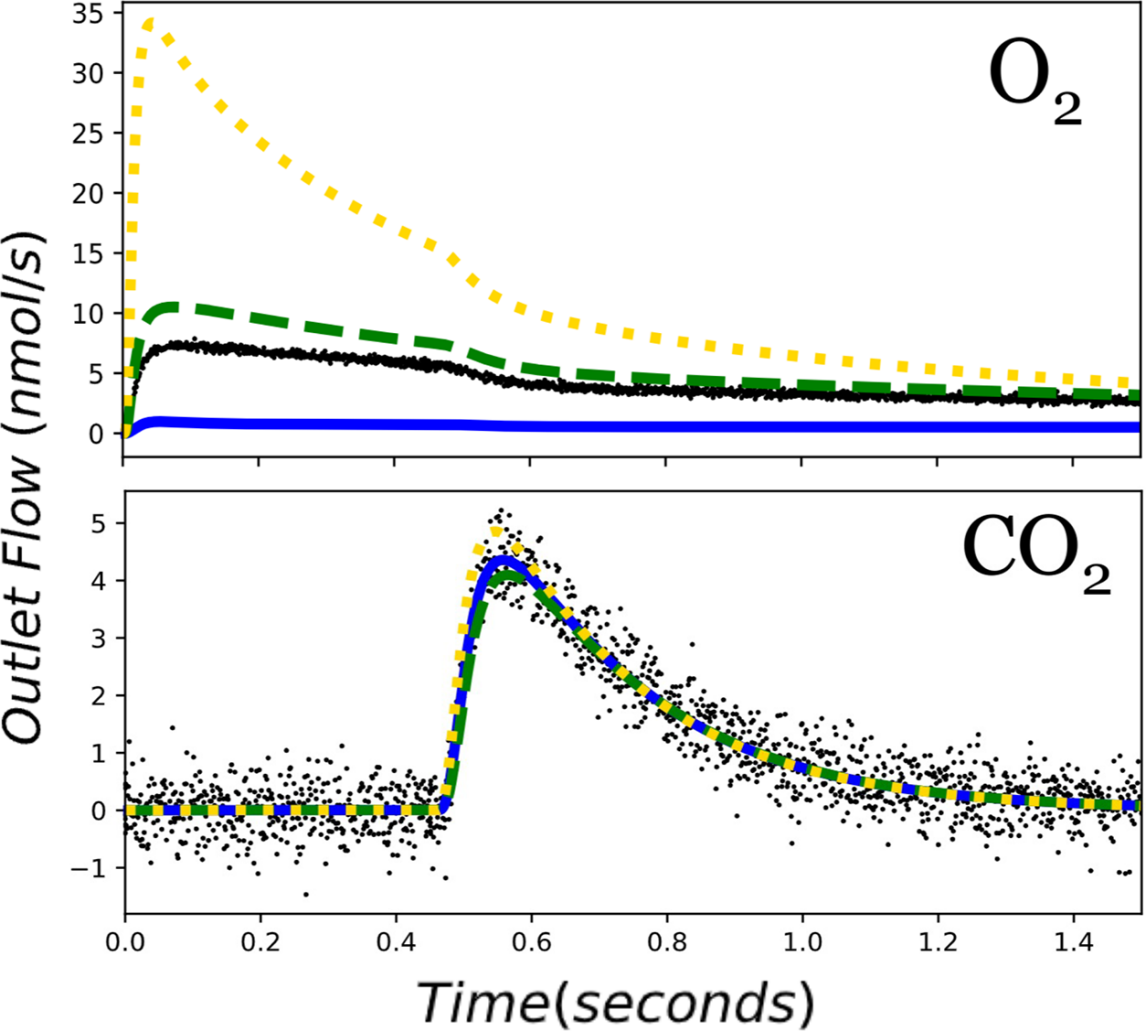
Visual of the differences observed between mechanism 1 (solid blue), mechanism 2 (dashed green), and mechanism 3 (dotted yellow) for the outlet flux of oxygen and carbon dioxide. Propane, propene, and water had agreements similar to those found in the carbon dioxide subplot above, while oxygen was the only graph with significant degrees of divergence. Mechanism 2 was found to agree most with the experimental data (black dots) and was quantitatively confirmed by the BIC values.

The same grid search approach over different experimental conditions is applied as previously introduced for MBDoE for precision. However, in this case, there is no parameter estimation or uncertainty quantification; the goal is to maximize the difference between each of the outlet fluxes of each mechanism. When running this analysis (i.e., calculating an explicit divergence criterion for each experimental condition in the grid search using eq 19), we found the set of conditions that led to the highest divergence to be 2 nmol propane and oxygen, a propane delay of 0.45 s, and a temperature of 700 K. All fluxes, with the exception of oxygen, were found to agree with the experimental data reasonably well. For this reason, only oxygen and carbon dioxide are presented in Figure 5. In the oxygen flux subplot, mechanisms 1 and 3 (solid blue and yellow, dotted line, respectively) are clear outliers, and the BIC is consistent with these mechanisms being less likely. Mechanism 2 (dashed, green line) falls within a similar window of outlet flux values. Mechanism 2 agrees more with the simulated experimental data and has a lower BIC, which provides evidence that it is the likely mechanism. These results indicate that TAP experiments are capable of discriminating between models with different active site configurations, even when the underlying kinetic parameters are very similar, and that MBDoE is an effective route to identifying which TAP experimental conditions are expected to provide the most discrimination between different candidate mechanisms.

## Discussion

### Efficacy of MBDoE for Precision

**Figure 6:**
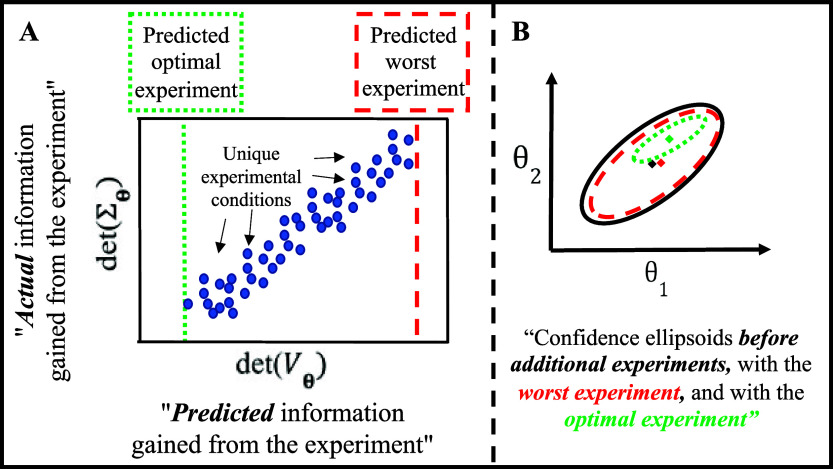
Conceptual representation of the results of a MBDoE for precision analysis, where each dot in (A) shows a unique experiment (i.e., a particular combination of pulse intensities, delays, and temperature found in the grid search). The correlation between predicted information gain (determinant of the inverted Fisher information matrix in D-optimal design) vs the actual information gain (the determinant of the covariance matrix for refitted model in D-optimal design) for a given parameter reveals the ability of MBDoE to identify the optimal experiment and the impact of using the optimal experiment on the actual information gained. This is more easily understood in panel (B) in the case of two parameters being explored through confidence ellipsoids, where the smaller confidence ellipsoid will align with the left most point in panel (A).

The results section shows that the experiments selected by MBDoE reduce the uncertainty of fitted parameters. However, the efficacy of the MBDoE over random experimentation is not clear. To provide a more rigorous evaluation of the performance of MBDoE, we explored the correlation between predicted and actual information gained, where the predicted information is defined by the determinant of the inverse Fisher information matrix, and the actual information is defined by the determinant of the covariance matrix after refitting the model.^46^ This is conceptually presented in Figure 6. Importantly, the Fisher information matrix is available without generating additional synthetic experimental data, while the covariance matrix requires experiments to be run/simulated. Thus, the comparison between predicted and actual information is only practical where (synthetic) experimental data can be easily generated and is used here to analyze the performance in a simulated scenario where this is possible.

The correlation in these graphs indicates the accuracy of the predicted information, while the difference between the minimum and maximum actual information indicates the influence of the experimental conditions on a given parameter; therefore, these graphs provide a convenient visual approach to evaluating the efficacy of MBDoE for precision. We used this approach to evaluate the choice of optimality criterion, comparing predicted vs actual information for D-, E-, and A-optimal criteria. The results, as shown in the Supporting Information, show that the correlation is strongest for the D-optimal criterion. For this reason, we focus on D-optimal experiments in all subsequent analyses.

**Figure 7:**
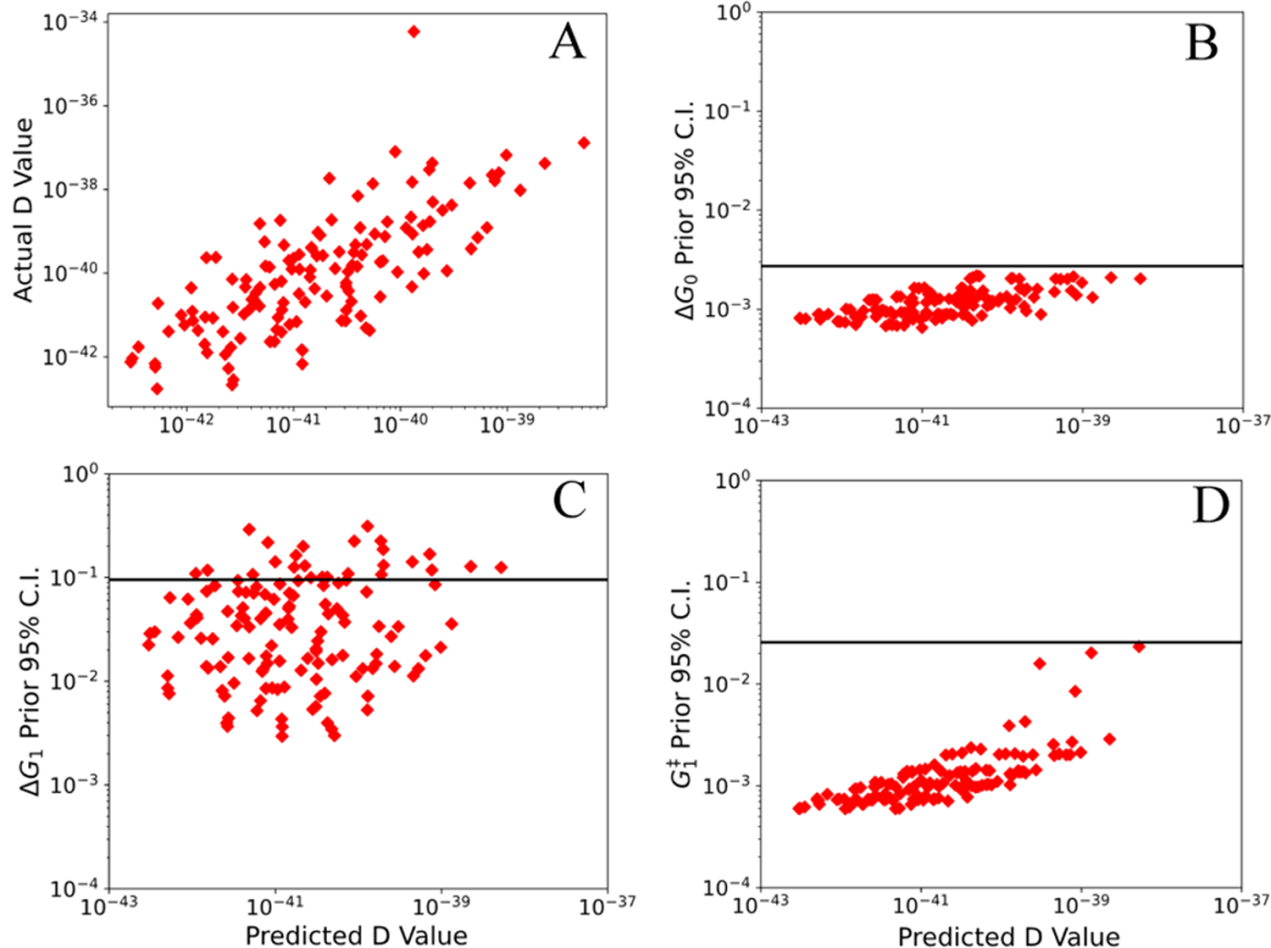
Predicted information (predicted *D*-value) gain compared to the actual information gained (actual *D*-value), as well as the confidence intervals for the parameters fitted in the system, for the second experiment. Each red point represents a different set of experimental conditions evaluated over the entire grid search, and the black horizontal line represents the confidence interval found after the first experiment.

The trends in the predicted information and the actual information after the first experiment are presented in Figure 7. Figure 7A shows the predicted D-optimal compared with the actual D-optimal (or determinant of the covariance matrix). There is a clear correlation between high and low D-optimal values, indicating that the determinant criterion should be a good predictor of parameter confidence interval reductions. Figure 7B shows the confidence intervals around the refitted values of Δ*G*_0_, or the free energy of the adsorption of propane. There is a reasonable correlation between predicted and actual *D*-values, and the D-optimal experiment outperforms the majority of the competing experimental designs (approximately 96%). Although the lowest confidence interval is not observed at the lowest predicted *D*-value, the overall reduction in the uncertainty of Δ*G*_0_ is relatively small for all experiments.

The activation of adsorption of oxygen (*G*_1_^‡^) was explored in Figure 7D and the uncertainty reduction found in Δ*G*_0_ is again observed. In this case, some experiments perform far worse than others, although no experiments lead to an increase in the uncertainty. The unfavorable experiments all fall at high predicted *D*-values and would be rejected. The uncertainty reduction in *G*_1_^‡^ is also larger than most other parameters, reducing by more than an order of magnitude from the prior experiment. This again highlights the fact that the MBDoE approach performs differently depending on which parameter is being investigated.

Next, in Figure 7C, the free energy of oxygen adsorption (Δ*G*_1_) was explored. Unlike Δ*G*_0_ and all other parameters, there is no trend in this distribution, and the “optimal experiment” leads only to a moderate reduction in the uncertainty. However, there are also examples of experiments where the confidence interval increases, indicating that while the D-optimal experiment may not be optimal, it is a significant improvement over randomly selecting experimental conditions. On the other hand, some experiments lead to significantly more reduction in uncertainty than the predicted optimal experiment, indicating that it is possible to further reduce the uncertainty on this parameter, but that the MBDoE fails to identify the optimal experiment for this parameter. We hypothesize that this occurs due to the low sensitivity (high error bar) of Δ*G*_1_, which causes the MBDoE to favor improvement of parameters that are already well-determined; we revisit this issue later in the section.

The evaluation of other parameters—propane adsorption (Δ*G*_2_) and activation energies for propane dehydrogenation, propane combustion, and propene combustion (*G*_3_^‡^, *G*_4_^‡^, and *G*_5_^‡^)—is also analyzed using the same technique. The results are shown in the Supporting Information, and they are largely consistent with the findings for Δ*G*_0_. A reasonably strong correlation is observed between the predicted and actual *D*-values, but the reduction in uncertainty is relatively small (<1 order of magnitude).

A similar analysis of predicted and actual *D*-values and confidence intervals for all parameters was also performed for the second iteration of MBDoE. The results, as shown in the Supporting Information, show a much lower correlation between actual and predicted *D*-values and significantly less reduction in uncertainty for the optimal experiment. Although some parameters show a slight decrease in uncertainty, most remain the same. There are also more examples of experiments that lead to increased uncertainty for many parameters that could be a result of some inaccurate parameter estimates. These findings suggest that there is little value in additional experiments from the MBDoE, although repeated experiments could help reduce uncertainty more gradually. This is consistent with the conclusion that the iterative MBDoE workflow shown in Figure 2 converged after two experiments.

### MBDoE for Precision of Specific Parameters

The results indicate that there is a lower limit to the precision that can be obtained by the standard MBDoE for the precision workflow proposed in Figure 2. This may be problematic in the case where the uncertainties on one (or more) parameter(s) of particular chemical interest are not sufficiently well-determined after the workflow converges.

The MBDoE for precision is typically used to reduce the uncertainty in all parameters simultaneously.^46^ Here, we propose a revised workflow that isolates a specific parameter and focuses on the parameter with the highest degree of uncertainty, in this case Δ*G*_1_. We hypothesize that focusing on this parameter will lead to a more precise estimate of other parameters as well, since it will reduce the overall uncertainty in the system.

**Figure 8:**
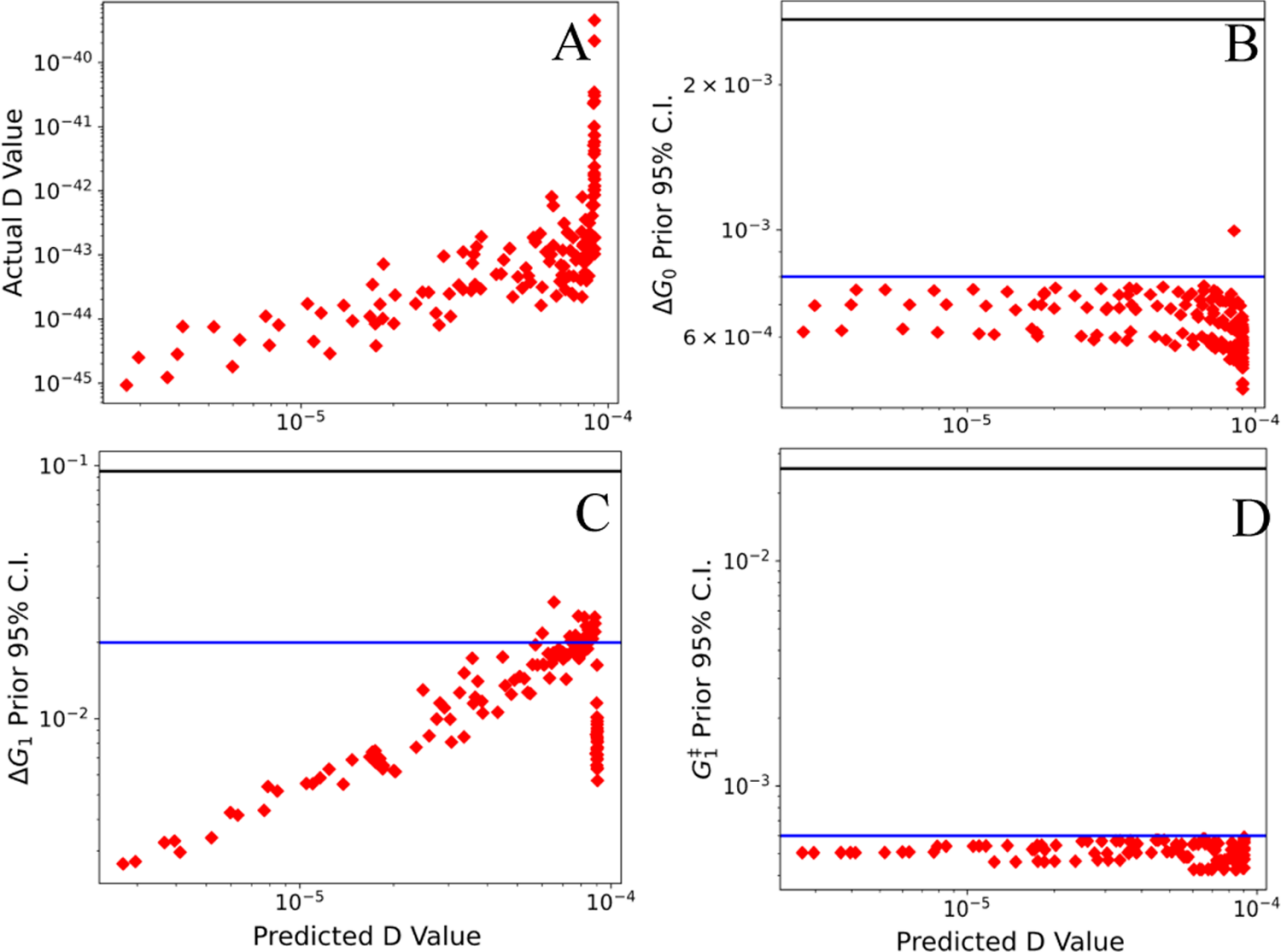
Predicted information (*D*-value) gain compared to the actual information gained (*D*-value), as well as the confidence intervals for the parameters fitted in the system for the third experiment using only the parameter with the highest uncertainty in the design. Each red point represents a different set of experimental conditions evaluated over the entire grid search, while the black horizontal line represents the confidence interval found after the first experiment, and the blue line represents the confidence interval found after the second experiment.

To test this, we performed the MBDoE for precision, with only the Δ*G*_1_ value and uncertainty included in the grid search. We then analyzed the impact of this selection criterion on the actual information gained (optimizing all parameters at the experimental conditions) and confidence intervals for each parameter in the system. The results of this analysis are presented in Figure 8. The *D*-value for Δ*G*_1_ (Figure 8A) shows a strong linear trend between the predicted optimal experiment and the true optimal experiment for most *D*-values, with a lack of correlation at high predicted *D*-values.

As expected, the uncertainty for Δ*G*_1_ (Figure 8C) is strongly correlated with the predicted *D*-value, with the optimal predicted experiment substantially reducing the uncertainty on Δ*G*_1_ by more than 1 order of magnitude. The correlation between the *D*-value and the uncertainty for other parameters is much weaker, and experiments that were found to perform the worst for Δ*G*_1_ at times led to the highest reduction in the uncertainty for other parameters. Specifically, Δ*G*_0_ (Figure 8B) and *G*_3_^‡^ (shown in the Supporting Information) had the lowest uncertainty for experiments that were predicted to be the worst for defining Δ*G*_1_, although other parameters (e.g., Δ*G*_2_ and *G*_4_^‡^) had significantly increased uncertainty for the worst predicted experiment (shown in the Supporting Information). These findings reveal that there can be anticorrelation between the optimality conditions for different parameters, providing insight into why the D-optimal design for all parameters simultaneously fails to systematically reduce the uncertainty on all parameters beyond a certain point.

On the other hand, the predicted optimal experiment for Δ*G*_1_ does lead to some reduction in the uncertainty for all parameters, and the resulting model has parameters that are more accurate and precise than the model where all parameters are included. These results indicate that focusing the DoE on a single poorly determined parameter is an effective strategy to reduce the uncertainty of that parameter, especially in the case that the full design of experiments does not significantly reduce uncertainty.

### Analysis of MBDoE for Divergence

**Figure 9:**
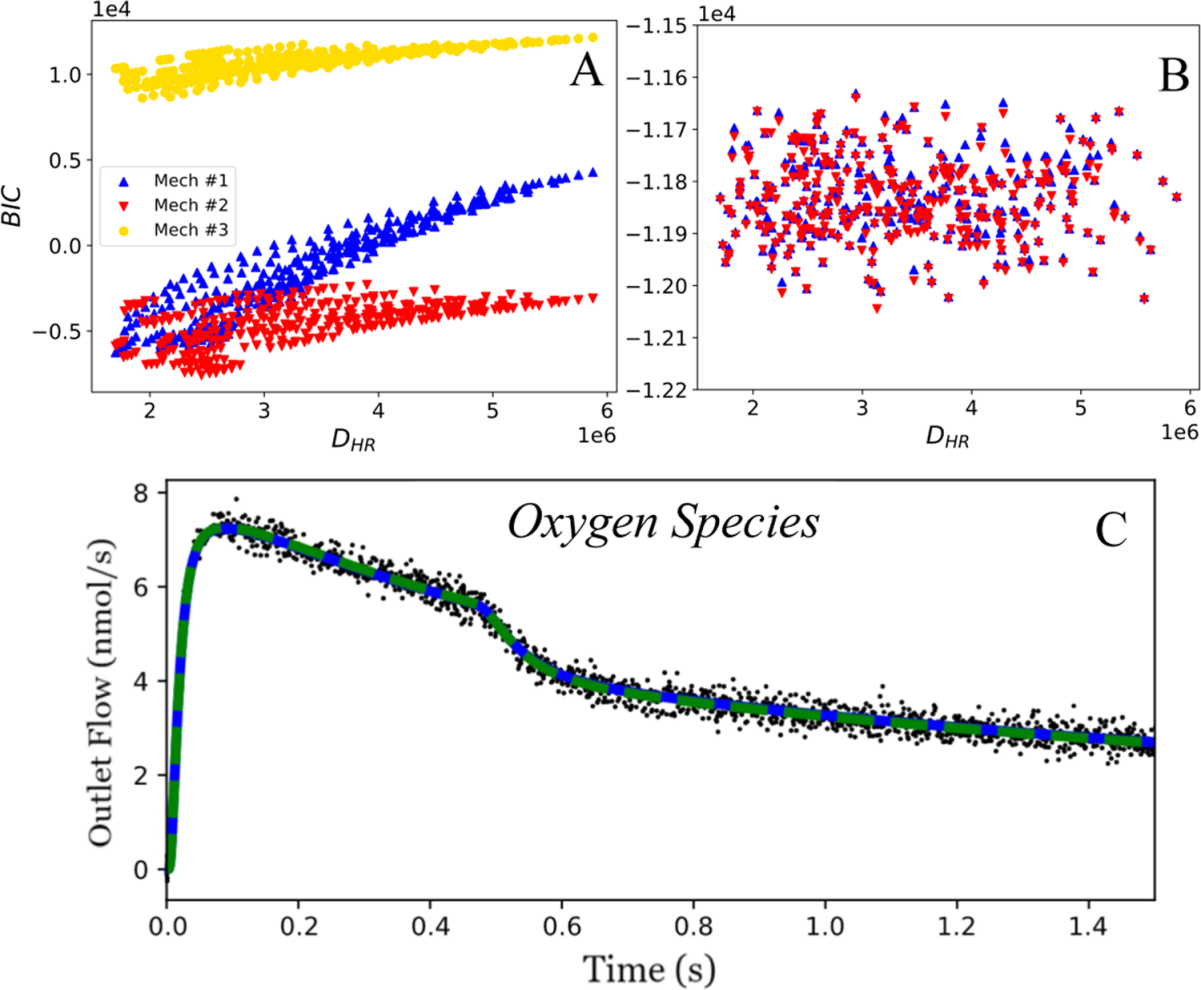
(A) Predicted divergence compared with the BIC value for each mechanism. At low predicted divergence values, it is more challenging to discriminate between mechanisms 2 and 3. At higher divergence values, mechanism 2 is highly favored (as visualized in Figure 5). Following optimization, the ability to adequately discriminate between mechanisms 1 and 2 is lost. Both mechanisms have similar BIC values (B) for each of the considered experimental conditions, with the outlet flow of oxygen (C) overlapping significantly.

As with MBDoE for precision, we want to confirm that we are observing a trend between the predicted divergence and the ability to find evidence of discrimination between the mechanisms. We performed a similar analysis as in the case of precision, simulating the experiments under all predicted conditions. In this case, we compare the predicted Hunter–Reiner divergence (eq 19) with the BIC calculated for the various mechanisms compared to each experiment. Since the BIC can provide evidence for the preference of a mechanism, there should be a correlation between the predicted maximum difference between each mechanism (i.e., the Hunter–Reiner divergence) and the difference in BIC values between the proposed mechanisms. The parameters are not reoptimized, and the spread in the BICs occurs due to the slight perturbations in kinetic parameters between experiments (as described in the Methodology section). This analysis is presented in Figure 9A. In all cases, the BIC of mechanism 3 is much higher than that of mechanisms 1 and 2, indicating that MBDoE is not necessary to discriminate between mechanism 3 and mechanisms 1 and 2. However, at low values of the mechanism divergence, there is a strong overlap between the BIC values for mechanisms 1 and 2, indicating that under these conditions, it is not possible to determine which mechanism is consistent with the data. However, higher divergence values lead to significant differences in BIC values between mechanisms 1 and 2. This shows that TAP experiments are able to differentiate between different types of single and multisite mechanisms even when the underlying kinetic parameters are very similar. It also reveals that MBDoE for divergence is necessary to distinguish between subtly different multisite mechanisms, while arbitrary experiments are sufficient for more distinct mechanisms such as single vs multisite mechanisms.

### Simultaneous Determination of Kinetic Parameters and Mechanism

In the above analyses, it was assumed that the mechanism was known, and the parameters were fitted and refined (precision), or that the parameters were known, and the experiments were used to differentiate between different mechanisms (divergence). In practice, it is often the case that both the kinetic parameters and the reaction mechanism are unknown. Thus, in a realistic scenario, it would likely be necessary to combine these two workflows. For example, in the case of mechanism discrimination, each different mechanism might be refitted to the experimental data.

To evaluate the combination of the proposed divergence and precision workflows, we reoptimize mechanisms 1 and 2 to the experimental data generated during the grid search for model divergence. This analysis is presented in Figure 9B. Ideally, there would be some correlation between the predicted divergence and the difference between the BIC values for the two different mechanisms. It is clear that after reoptimization, the BIC is not correlated to the divergence, and discrimination between the two mechanisms is not possible under any of the experimental conditions explored. There are some experiments in which small differences can be observed, but they are scattered randomly throughout the divergence and are too small to draw any strong conclusions. Prior optimal experiments based on the divergence criterion are also visually compared after reoptimization (Figure 9C) and are essentially indistinguishable. These results suggest that the convolution of parametric and structural uncertainty presents a significant challenge since it is not possible to distinguish between mechanisms if the parameters are obtained by fitting to the kinetic data.

The two components of this investigation, parameter fitting (precision) and mechanism discrimination (divergence), are often combined into a single workflow.^46^ A single mechanism is identified using discrimination approaches or, more commonly, prior literature and intuition. Next, the parameters of this mechanism are fit and can be refined using MBDoE. Our current results show that this approach becomes problematic when the flexibility in the mechanism increases, causing a scenario where complex mechanisms can describe a broad range of experimental data if parameter optimization is performed, even for transient kinetic data sets. This presents a challenge for the field, which could potentially be overcome with additional data in various forms. For example, multipulse (state-altering) experiments, or more data from experiments under diverse conditions, could be included to further constrain the parameters and mechanisms.^36^ Similarly, spectroscopic data can provide direct information on surface species, as well as on the structure of the catalytic material, potentially enabling better differentiation between different mechanisms and active site structures.^32^ Moreover, improvements in the optimization techniques may help alleviate the problem by providing more accurate and efficient estimates of parametric uncertainty.^70−72^ Another approach is to explore alternative experimental design paradigms. Bayesian experimental design^19,20^ and robust information gain^73^ may enable the design of experiments that more effectively decouple parametric and structural uncertainty. Alternatively, modifications of the current workflow, such as reversing the order of mechanism divergence and precision and introducing additional experimental techniques, could also show promising results. For example, the proposed mechanisms could be refined using TAP experiments and then validated (or discriminated between) using high-pressure experiments.^9,74^ In addition, theoretical work to understand the limits of what parameters and mechanisms can be reliably identified from experimental data may help constrain candidate mechanisms to reduce flexibility and provide more efficient differentiation between candidate mechanisms.^75,76^

## Conclusions

Transient kinetic experiments provide investigators with dense data sets that can elucidate complex reaction mechanisms. The TAP reactor, a low-pressure transient kinetic method with millisecond time resolution, is particularly well-suited to deconvolute the intrinsic kinetics of catalytic materials.^36,37^ The information gained from these experiments can be heavily dependent on the choice of initial conditions (e.g., pulse intensities, pump/probe delays, reactor temperature, etc.). Since no quantitative approach for identifying optimal TAP experiments has previously been introduced, we explore the use of MBDoE for precision and divergence in a synthetic oxidative propane dehydrogenation case study.

The proposed workflow for selecting experiments and optimizing parameters is introduced and built around the Fisher information matrix, which combines a current understanding of the parameters (covariance) and the predicted gain in information from a new experiment (the dynamic sensitivity matrices). We found that the MBDoE for precision is capable of identifying the most informative experimental conditions, resulting in an increase in confidence for most of the parameters. However, the reduction in uncertainty saturated after two experiments and one parameter still had a significant confidence interval. A variation in the design criteria provided a route to further reduce the uncertainty of this parameter as well as others, suggesting a clear path to refine the precision of fitted parameters through MBDoE.

Structural uncertainty, which is observed in catalytic studies in the form of active site configurations or inclusion of reaction steps, was also explored in TAP through MBDoE. The findings indicate that TAP is capable of differentiating between even subtle differences in reaction mechanisms, including different types of single and multisite models. However, this differentiation relies on prior knowledge of the rate parameters that control the kinetics, and we find that it is not possible to differentiate between these subtly different mechanisms if parameter optimization is not performed for each case.

Additional complications may also arise when working with real experimental data. For example, certain molecules, such as water, may be difficult to observe or have a particularly high degree of uncertainty. There is also often significant uncertainty associated with the characterization of the solid catalyst and catalytic surfaces. Therefore, including the effects of missing or incomplete information in the design of experiments is expected to be an important area of future work. Generally, we expect that the MBDoE workflow will be an effective tool for guiding TAP experiments with quantitative feedback for both parameter refinement and model discrimination. Future implementations involving state-altering experiments and spectroscopic data as well as applications to non-Knudsen operating conditions could make this a more powerful tool, but improvements to efficiency and automation will be necessary.

## Supporting Information Available

Parameter sensitivities; initial conditions; variable definitions; and details on running the TAPSolver code (PDF)
