# Supplementary material for: Model-Based Design of Experiments for Temporal Analysis of Products (TAP): A Simulated Case Study in Oxidative Propane Dehydrogenation
Source: Ind Eng Chem Res. 2024 Mar 11;63(11):4756–70. doi: 10.1021/acs.iecr.3c03418 (PMC10958505; doi:10.1021/acs.iecr.3c03418)
Supplement: Supplementary file 1 — ie3c03418_si_001.pdf [file ie3c03418_si_001.pdf]

Supporting Information: Model-based design of experiments for temporal analysis of products (TAP): A simulated case study in oxidative propane dehydrogenation

Adam Yonge,[†] Gabriel S. Gusmão,[†] Rebecca Fushimi,[‡] and Andrew J. Medford[*]

†*College of Engineering, Georgia Institute of Technology, Atlanta, Georgia, 30332 USA*

‡*Catalysis and Transient Kinetics Group, Idaho National Laboratory, Idaho Falls, Idaho, 83415 USA*

E-mail: ajm@gatech.edu

Model parameter sensitivities for MBDoE for precision

To narrow the parameters explored during the MBDoE for precision analysis, we looked at the sensitivity of all parameters to determine which are identifiable. Not all parameters will be experimentally observable due to their extreme values (i.e. if the energy is too high or too low, varying the energy in the model will not alter reaction rates). Using the initial parameter guesses and the seven-parameter fit, we show all the parameter sensitives in Table S1. The parameters excluded from the analysis have sensitives at and below 1e-3, whereas the included parameters had higher values. For this reason, we performed the analysis with these seven parameters.

Table S1: The sensitives of the parameters found with the initial parameter guesses and the final fit of the seven parameter optimization (the local minimum) .

Parameters	Initial Sensitivity	Local Minimum Sensitivity
ΔG_0	-4.92e5	7.64e-1
$G_0^{\ddagger}$	-1.80e-3	1.20e-5
ΔG_1	3.28e5	1.15e-2
$G_1^{\ddagger}$	-5.97e1	-1.31e-1
ΔG_2	-1.53e4	7.31e-2
$G_2^{\ddagger}$	3.55e-6	2.42e-6
ΔG_3	-3.08e1	1.07e-3
ΔG_4	4.45e-29	-1.96e-33
ΔG_5	4.51e-24	2.70e-25
$G_3^{\ddagger}$	-1.61e5	1.04e0
$G_4^{\ddagger}$	-4.99e4	1.08e-1
$G_5^{\ddagger}$	-1.12e5	1.99e-1

Correlation between predicted D and actual D criteria

We compared different methods for distilling the Fisher information matrix and calculated covariance matrices as the A (trace), D (determinant), and E (eigenvalue) criteria. Although each is used in the literature, we observed the strongest correlation for the D-optimal criteria and therefore use it for MBDoE.

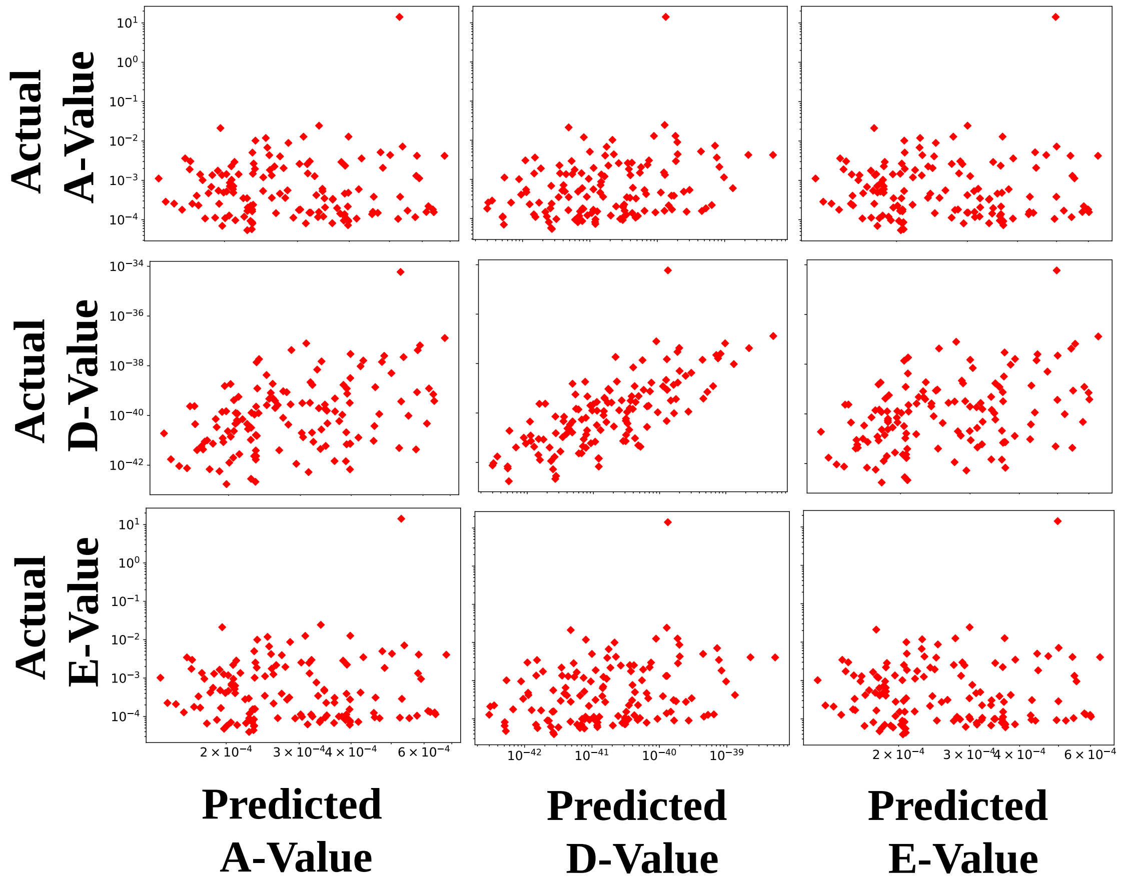

Figure S2: The predicted A, D, and E criteria for designing experiments were compared to their actual A, D, and E criteria for each of the possible experiments. The D criteria, which stands for the determinant, is the only criteria that had a strong correlation and, for that reason, is the only criteria thoroughly explored in this paper.

Initial conditions used during the precision refinement process

The MBDoE approach for precision used an initial, arbitrary experiment (1st experiment in Table S2), followed by three additional experiments. The 2nd experiment is the first experiment predicted using MBDoE, while the 3rd experiment is the second experiment predicted using MBDoE. The *Alt.* experiment involves the adjusted approach for designing the experiments involving only the most uncertain parameter.

Table S2: The experimental conditions used to constrain the parameters. Experiment 1 was selected arbitrarily, while experiments 1 and 2 were selected through MBDoE for precision.

	C_3H_8 Intensity (nmol)	O_2 Intensity (nmol)	O_2 Delay (s)	Temperature (K)
1st Experiment	1.0	1.0	0.00	700
2nd Experiment	2.0	2.0	0.60	650
3rd Experiment	2.0	2.0	0.15	650
Alt. Experiment	2.0	2.0	0.60	700

MBDoE experiment selection correlation

We observed the performance of the MBDoE for several parameters in the oxidative propane dehydrogenation reaction, but focused only on three parameters in the primary text. For this reason, we provide the remaining plots in Figures S3 through S5 for the second experiment selection, third experiment selection, and the alternative third experiment selection.

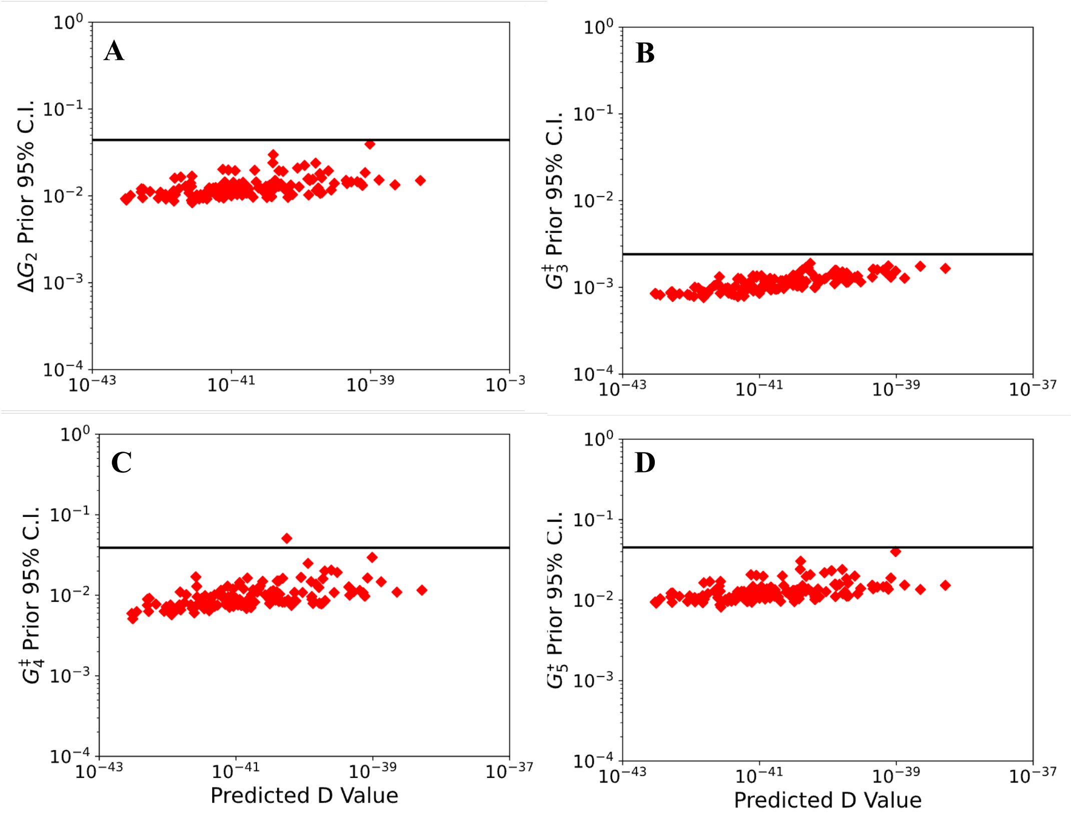

Figure S3: The predicted improvement to kinetic understanding using D-optimality for additional parameters fitted following the first experiment.

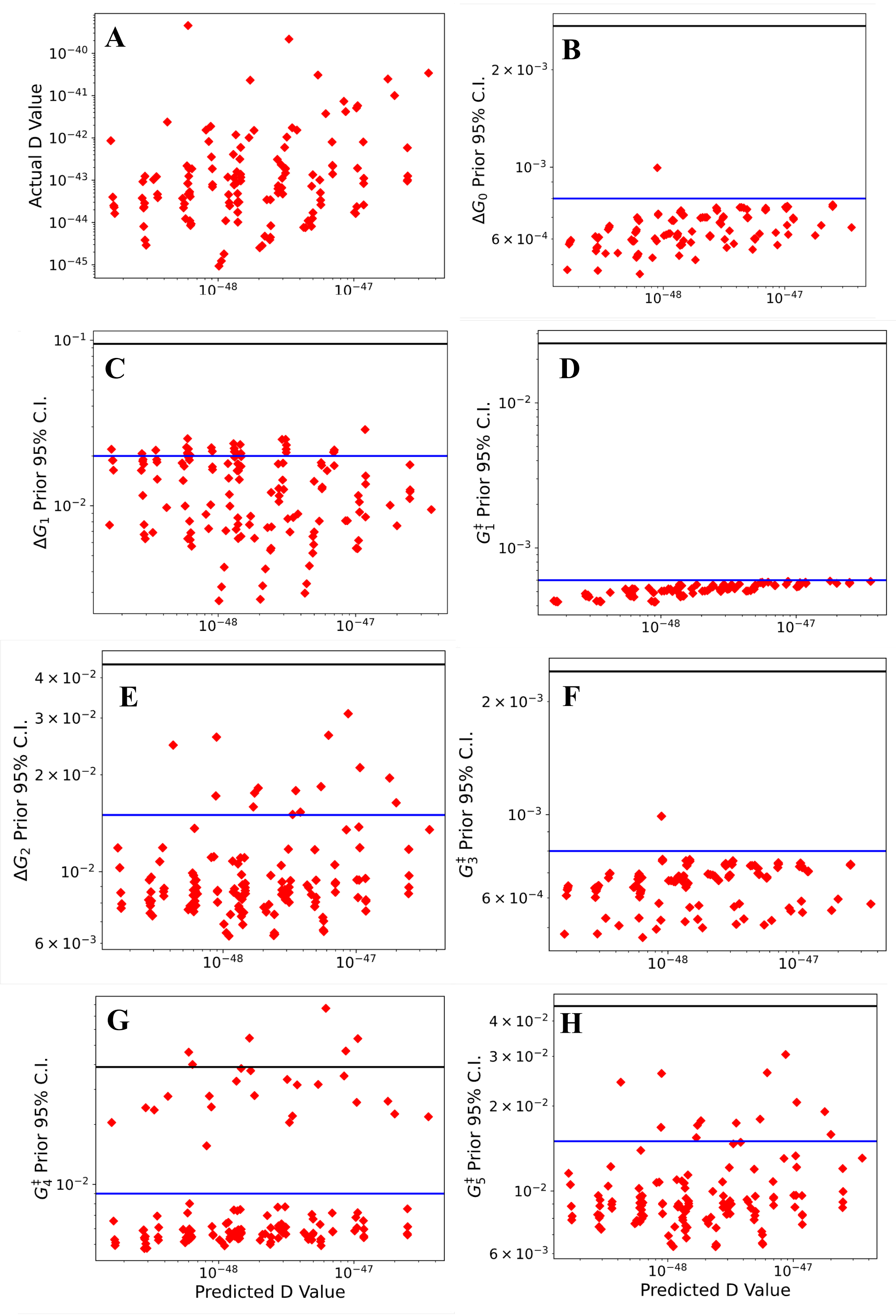

Figure S4: The predicted improvement to kinetic understanding using D-optimality for all parameters following the second experiment.

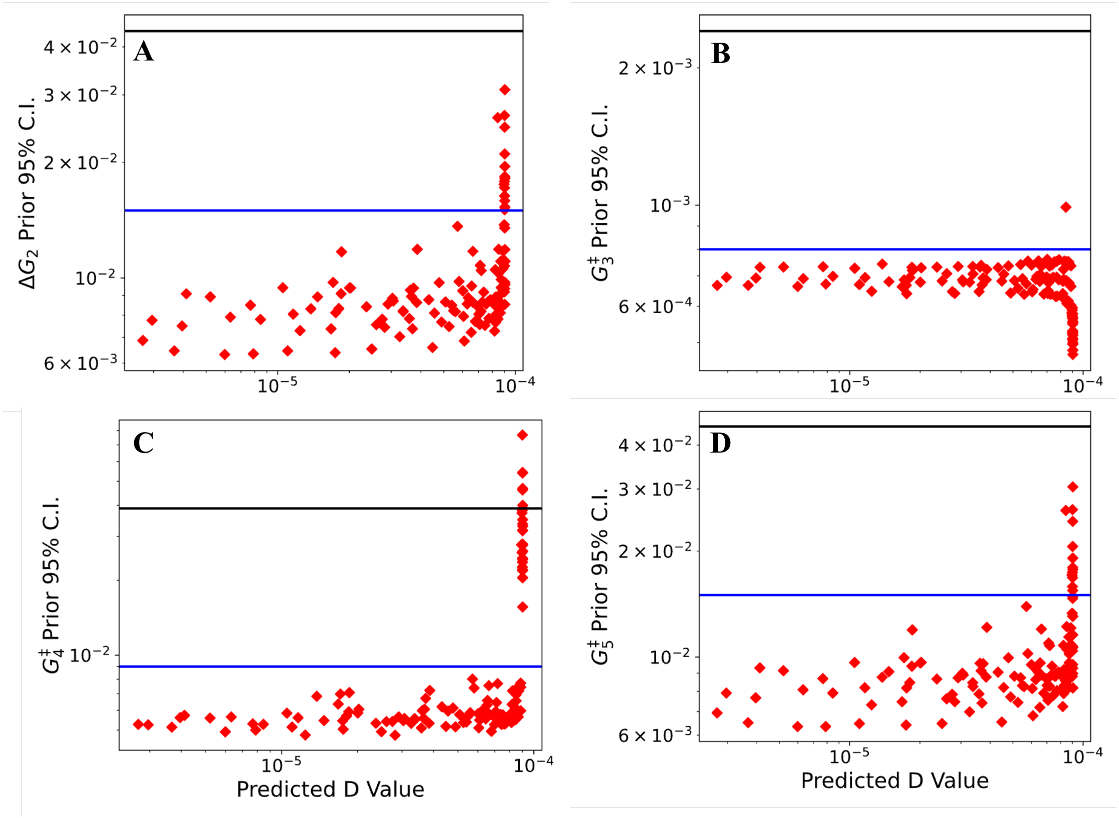

Figure S5: The predicted improvement to kinetic understanding using D-optimality for additional parameters fitted following the third experiment.

MBDoE experiment selection correlation

Although we only considered mechanism two as the black box experiment in the primary text, we also explored the use of mechanism 1 and 3 as the black box experiment (shown in the top and bottom plots of Figure S6). These experiments showed clear divergence between each other, so we focused on mechanism 2, which has non-trivial divergence for some experiments (e.g. there exist experiments for which it is not possible to clearly distinguish mechanism 1 and 2 based on BIC).

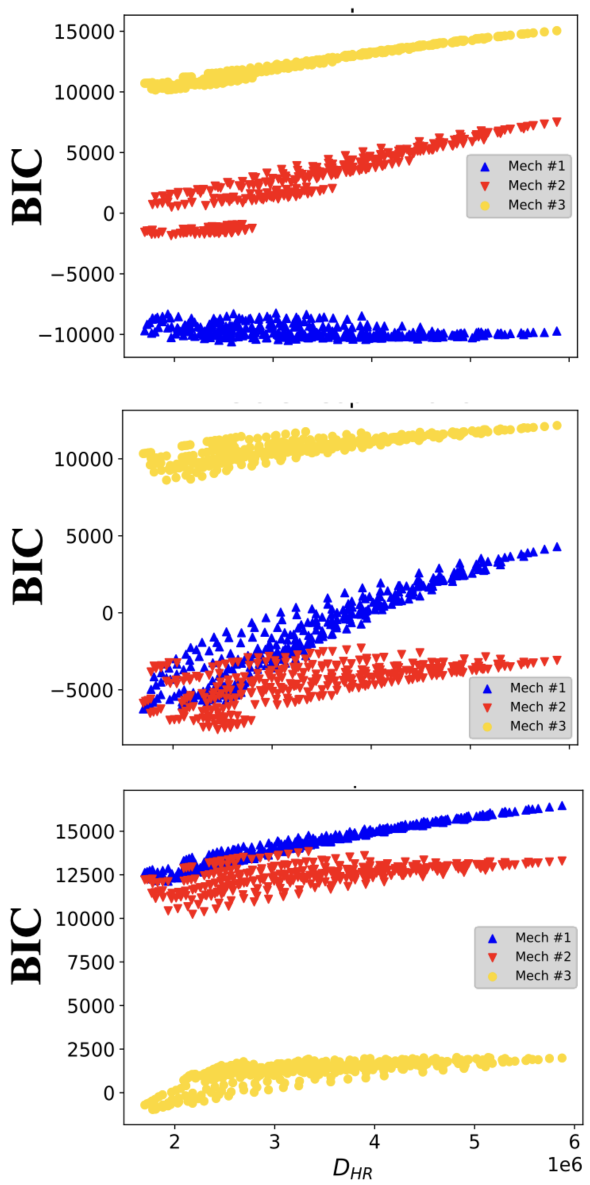

Figure S6: Divergence plots for the three oxidative propane mechanisms provided in the primary text. The top, middle, and bottom plots represent divergence when the experimental data is generated using mechanism 1, 2, and 3, respectively.

Running TAP simulations

All simulations and analyses were performed using the internally developed and publicly available TAPsolver software. We have previously hosted software workshops on TAPsolver and have provided the associated Jupyter notebooks, as well as documentation, on how to write and interpret TAPsolver code. We also include example scripts used in the MBDoE analysis in the same directory as the notebooks. We have frozen the version of the TAPsolver branch used to perform the simulations in Zenodo, as well as the raw simulated data used in the analysis (sensitivities, simulations, fits, uncertainties, etc.).

Links to the code are provided below:

Zenodo link: `https://zenodo.org/records/10456211`

Github repo: `https://github.com/medford-group/TAPsolver/tree/mbdoe_publication`

Variable names

Variable	Meaning
A_{opt}	A optimality
BIC	Bayesian information criteria
$\mathbf{c}$	Concentration vector
$\dot{\mathbf{c}}$	Time dependent concentration vector
c_i	Concetration of gas i
δ	Dirac delta function
$\mathbf{d}$	Diffusion coefficient vector
D_i	Diffusion coefficent of gas i
D_{opt}	D optimality
D_{ref}	Reference diffusion coefficient
$D_{TAP,HR}$	Hunter-Riner divergence criteria for TAP
E_{opt}	E optimality
ε	Void fraction
$\varepsilon_{n,s}$	Model residual at experiment n and time step s
$\mathbf{f}$	Vector of gas fluxes
$\hat{\mathbf{f}}_{n,s}$	Simulated outlet flux at experiment n and time steps s
$\mathbf{f}_{n,s}$	Experimental outlet flux at experiment n and time step s
$G^{\ddagger}$	Free energy of activation
h_p	Planck constant
H_{θ}^{N}	Hessian matrix of all experiments N considered for the kinetic parameters of interest
i	Gas species
J^N	Objective function
k, l	Parameters of interest indices

k_b	Boltzmann constant
k_n	Number of kinetic parameters in the model
k_{tst}	Rate constant
L	Reactor Length
L_{CZI}	Catalyst zone inlet length
L_{CZO}	Catalyst zone outlet length
M	Total number of models
$\mathbf{M}$	Stoichiometric matrix
M_i	Mass of species i
M_{ref}	Reference mass
n	Specific experiment index
N	Experiment number total
N_{samp}	Sample size of data points
$\Omega_{n,s}$	Precision matrix of experiment n and time step s
P_i	Pulse intensity of species i
Q_i	Dynamic sensitivity matrix of gas i
r	Vector of individual reactions
R	Ideal gas constant
s	Time step index
S	Time step number total
$\hat{\sigma}_n, s$	Standard deviation of experimental noise at experiment n and time s
σ	Vector of standard errors of the parameters of interest
$\Sigma_{n,s}$	Inverse of the covariance experiment
Σ_θ^N	Covariance matrix around parameters of interest
t	time
$t_{i,p}$	Pulse delay of species i
T_{exp}	Experimental temperature

T_{ref}	Reference temperature
θ	Kinetic parameter of interest
u_i	Surface species concentration i
v_i	Initial concentration of surface species i
V	Inverse Fischer information matrix
x	Reactor length
$*$	Active site type 1
$\wedge$	Active site type 2

TOC Graphic

Some journals require a graphical entry for the Table of Contents. This should be laid out "print ready" so that the sizing of the text is correct.
Inside the `tocentry` environment, the font used is Helvetica 8 pt, as required by *Journal of the American Chemical Society*.
The surrounding frame is 9 cm by 3.5 cm, which is the maximum permitted for *Journal of the American Chemical Society* graphical table of content entries. The box will not resize if the content is too big: instead it will overflow the edge of the box.
This box and the associated title will always be printed on a separate page at the end of the document.
